# Diversity, environmental requirements, and biogeography of bivalve wood-borers (Teredinidae) in European coastal waters

**DOI:** 10.1186/1742-9994-11-13

**Published:** 2014-02-13

**Authors:** Luísa MS Borges, Lucas M Merckelbach, Íris Sampaio, Simon M Cragg

**Affiliations:** 1 Institute of Marine Sciences, School of Biological Sciences, Portsmouth University, Portsmouth, UK; 2 Helmholtz-Zentrum Geesthacht, Centre for Material and Coastal Research, Geesthacht, Germany; 3 Department of Biology, Centre of Molecular and Environmental Biology (CBMA), University of Minho, Braga, Portugal

**Keywords:** Teredinids, Shipworms, Sea surface temperature, Sea surface salinity, Life history, European coastal waters

## Abstract

**Background:**

Bivalve teredinids inflict great destruction to wooden maritime structures. Yet no comprehensive study was ever carried out on these organisms in European coastal waters. Thus, the aims of this study were to: investigate the diversity of teredinids in European coastal waters; map their past and recent distributions to detect range expansion or contraction; determine salinity-temperature (*S-T*) requirements of species; flag, for future monitoring, the species that pose the greatest hazard for wooden structures.

**Results:**

A total of nine teredinid species were found established in European coastal waters. Seven were considered cryptogenic, of unknown origin, and two were considered alien species. *Teredo navalis* and *Nototeredo norvagica* were the species with the widest distribution in European waters. Recently, *T. navalis* has been reported occurring further east in the Baltic Sea but it was not found at a number of sites on the Atlantic coast of southern Europe. The Atlantic lineage of *Lyrodus pedicellatus* was the dominant teredinid in the southern Atlantic coast of Europe. In the Mediterranean six teredinid species occurred in sympatry, whereas only three of these occurred in the Black Sea. The species that pose the greatest hazard to wooden maritime structures in European coastal areas are *T. navalis* and the two lineages of *L. pedicellatus*.

**Conclusions:**

Combined data from field surveys and from the literature made it possible to determine the diversity of established teredinid species and their past and recent distribution in Europe. The environmental requirements of species, determined using climatic envelopes, produced valuable information that assisted on the explanation of species distribution. In addition, the observed trends of species range extension or contraction in *Teredo navalis* and in the two lineages of *Lyrodus pedicellatus* seem to emphasise the importance of temperature and salinity as determinants of the distribution of teredinids, whereas their life history strategy seems to play an important role on competition.

*Teredo navalis* and *pedicellatus*-like *Lyrodus* species should be monitored due to their destructive capability. The two alien species may expand further their distribution range in Europe, becoming invasive, and should also be monitored.

## Background

The activity of bivalves of the family Teredinidae (shipworms) causes serious economic problems on wooden maritime structures [1], and on invaluable underwater cultural heritage [2]. Although no definite evaluation has ever been carried out Europe-wide, the estimated damage is of the order of millions of Euros per year [3]. Accounts of the damage caused by teredinids in Europe can be traced back to the writings of Greek (e.g. Theophrastus; 371–328 BC) and Latin (e.g. Ovid; 43 BC to 17 AD) authors [4]. However the first scientific studies were only carried out in Europe in the 18th century, when outbreaks of wood borers in the wooden dykes of The Netherlands caused catastrophic destruction [5]. Nowadays, the activity of these organisms is still of great concern in Europe [3,6-11] and was the subject of a recent “exploratory workshop on wood borers: new frontiers for European waters” [12].

Despite the economic importance of teredinids, a Europe-wide study on these organisms has never been carried out, and the exact number of established species in European coastal waters is still unknown. Furthermore, a review of the literature revealed scattered information with most studies being of relatively short duration at either single sites [13] or countries [14]. The important study by Roch [15] in the Mediterranean generated a wealth of data on the diversity and distribution of teredinid species, but it also included many synonyms and therefore the data should be used with caution. Although each of these studies reported several teredinid species occurring in Europe, there is a discrepancy in the number of species reported. For instance, Turner [16] considered six established species, whereas Nair & Saraswathy [17] reported nine, and Gofas and colleagues [18] reported 14 teredinid species.

There are several reasons for discrepancy mentioned above. The first is that some reported teredinid species were based on publications pre-dating the work of Turner [16], and synonyms are still listed as valid species (e.g. *Teredo utriculus*). Another reason is the fact that some species have been reported irrespective of whether found established or not in a sampled area. Moreover, teredinids are a particularly difficult group to identify from morphological features alone. In fact, identification of some teredinid species (e.g. *pedicellatus*-like *Lyrodus*) based solely on morphological characters is not sufficient to distinguish them [1,3,19]. Therefore, to improve ‘taxonomic resolution’ it is important that future taxonomic work on teredinids includes sequence data (mitochondrial and nuclear), in addition to morphology [1,3].

The distribution of teredinid species in Europe and the factors controlling their distribution is also poorly known. The distribution of marine organisms is known to be controlled by the interplay of a multitude of environmental and biological variables [20]. However, certain environmental factors are known to exert a dominant control over the natural distribution of species [21,22]. Temperature and salinity have been recognised to be the most important environmental determinants of abundance and geographical distribution of marine poikilotherms [23]. Indeed, several studies on teredinids have confirmed the importance of temperature and salinity in the physiology, distribution and activity of these organisms [8,14-17,24]. However, environmental requirements of temperature and salinity have not been investigated in the majority of teredinid species.

Climatic envelopes can be very useful for determining the environmental requirements of species. They commonly use associations between environmental variables and species distribution data (occurrence or abundance) to identify environmental conditions suitable to maintain populations of a species in a given area [21,22,25]. However, to infer the environmental requirements of species using climatic envelopes, high-resolution environmental data are required [26] in addition to high-quality occurrence data. Until recently, the global datasets of surface temperature and salinity had too coarse a resolution to resolve coastal areas adequately. Recent efforts have led to the development of BIO-ORACLE [20], a high resolution global oceanographic dataset. Furthermore, the output of high-resolution (regional) hydrographic models are increasingly being made available in publicly accessible databases, such as COSYNA [27], improving even further the resolution of coastal areas. These high-resolution datasets have the potential to stimulate the use of climatic envelopes, to infer environmental requirements and the potential distribution of many other coastal species.

Life-history patterns of teredinid species, when considered along with the environmental requirements, may also help to explain the distribution of species [16,28,29]. Larvae of oviparous species (larvae with external development) may be transported over great distances by currents [30] or in ballast water [31,32]. The adults and larvae of short and long-term larviparous (species which brood the larvae to the straight-hinge and pediveliger stages, respectively) may also be transported over great distances for instance in driftwood by currents [33]. Therefore, dispersing factors such as currents and human activities play a very important role in the actual distribution of species [31].

In our study we used a combination of field surveys, over a ten-year period, and a comprehensive review of the literature, as far back as 1900, to investigate the diversity of established teredinid species in European waters, including the occurrence of potentially invasive species. We used the occurrence data from our field surveys and from the literature to map the past (before 2000) and recent (since 2000) distribution of the species established in Europe. This made it possible to observe trends of expansion or contraction of species’ range. In addition, we developed a climatic envelope using the occurrence data coupled with data from a hybrid dataset, we compiled from high-resolution oceanographic datasets, to infer the salinity-temperature (*S*-*T*) requirements of teredinid species. Finally, we determined which teredinid species pose the greatest hazard to wooden maritime structures in Europe and flagged them for future monitoring.

## Results

### Diversity and distribution of established teredinids in European coastal waters

Teredinids occurred in 24 out of 32 sites surveyed between 2001 and 2011. They did not recruit to panels deployed at Reykjavik, Iceland; Gulf of Riga, Latvia; Island of Jürmo, Finland; Oban, Scotland; Swanage, Bournemouth and Lyme Regis, England.

**Figure 1:**
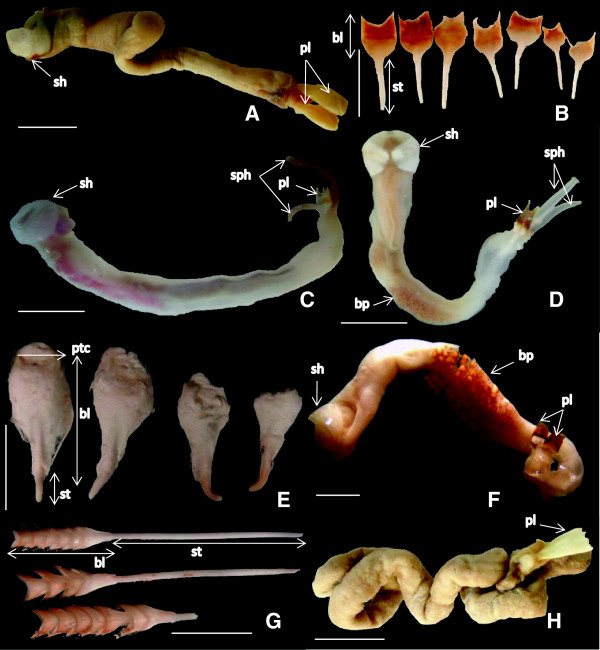
**Specimens and pallets of teredinids collected in the field surveys.** Specimens and pallets of teredinids collected in the field surveys: **A)** preserved specimen of *Nototeredo norvagica*; **B)** pallets of *Teredo bartschi*; **C)** fresh specimen of *Teredo navalis*; **D)** fresh specimen *Lyrodus pedicellatus* (Atlantic form); **E)** pallets of *Psiloteredo megotara*; **F)** preserved specimen *L. pedicellatus* (Mediterranean form); **G)** pallets of *Bankia carinata*; **H)** preserved specimen of *Teredothyra dominicensis*. Scale bar: complete specimens = 1 cm; pallets = 5 mm; sh-shell; pl-pallet; sp-siphon; bl-blade; st-stalk; pt-periostracum; bp-brood pouch.

A total of nine teredinid species were found established in European coastal waters, eight found in our field surveys and one only from previous studies. Of the nine species, six occurred in the Mediterranean, six in the Atlantic, three in the Black Sea and two in the Baltic Sea. *Nototeredo norvagica* (Spengler, 1792) (Figure 1A), *Teredo bartschi* (Clapp, 1923) (Figure 1B) and *Teredo navalis* Linnaeus, 1758 (Figure 1C) occurred both on the Atlantic and Mediterranean coasts of Europe.

A study by Borges and colleagues [3] revealed a genetic split between the Atlantic and eastern Mediterranean forms of the morphospecies *Lyrodus pedicellatus* (Quatrefages, 1849). Thus in present work we considered two lineages of *pedicellatus*-like *Lyrodus*, hereafter referred to as the Atlantic and Mediterranean forms. The Atlantic form of *L. pedicellatus* (Figure 1D), *Psiloteredo megotara* (Hanley, 1848) (Figure 1E) and *Teredora malleolus* (Turton, 1822) occurred in the Atlantic, while the Mediterranean form of *L. pedicellatus* (Figure 1F) *Bankia carinata* (Gray, 1827) (Figure 1G) and *Teredothyra dominicensis* (Bartsch, 1921) (Figure 1H) occurred in the Mediterranean. In our field survey in the Black Sea only *Teredo navalis* recruited to our panels, but recently a *pedicellatus*-like *Lyrodus* species and *Nototeredo norvagica* were also reported in the area [9]. *Teredo navalis* was the only species found in our test sites located in the Baltic Sea, but *Psiloteredo megotara* has been reported in the literature to occur occasionally in the area [14].

**Figure 2:**
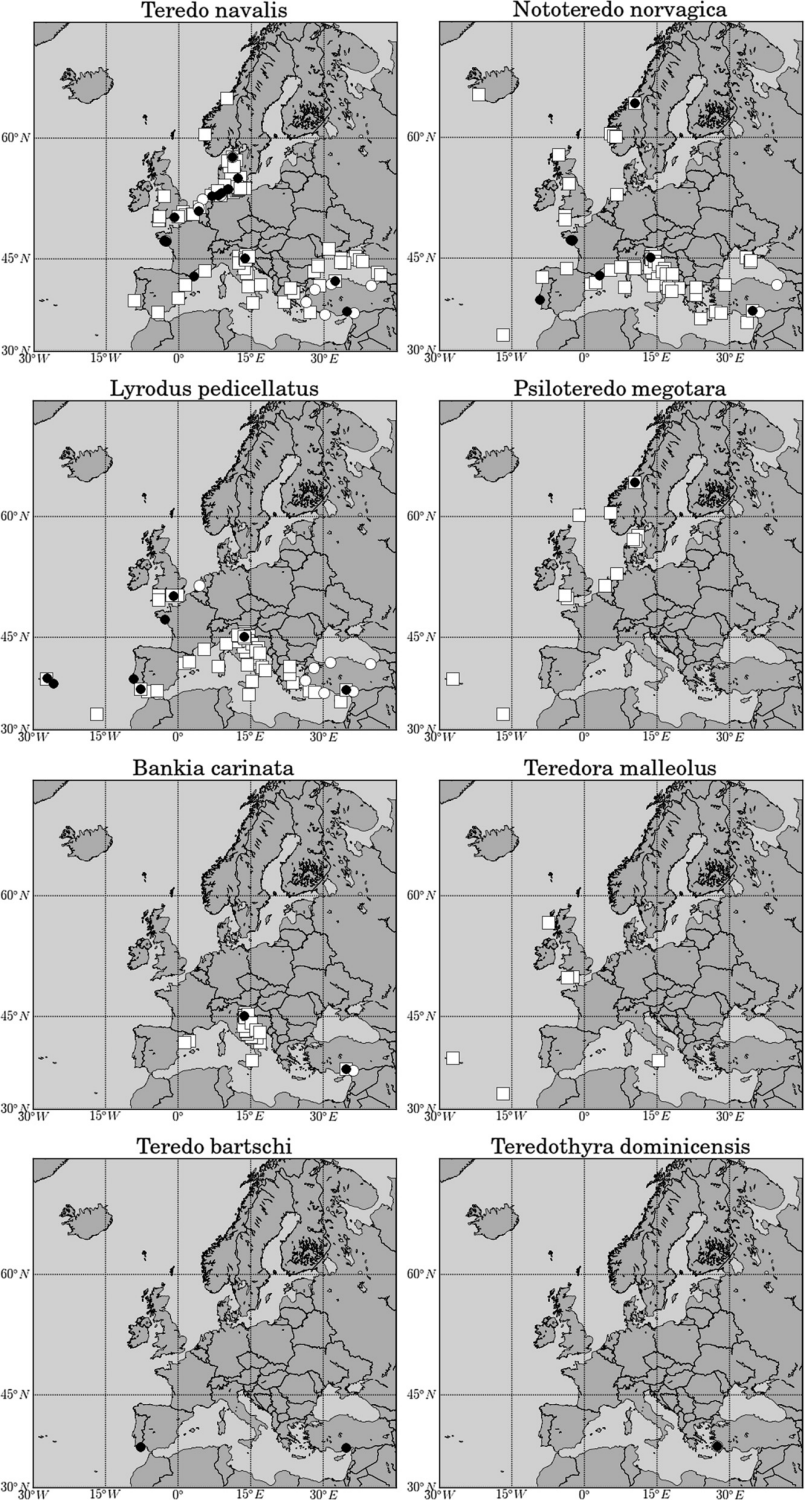
**Occurrence of teredinid species in European coastal waters.** Occurrence of teredinid species in European coastal waters. Squares represent data obtained from the literature before 2000; black circles represent data obtained from field surveys between 2001 and 2011; white circles represent data reported in the literature since 2000.

*Teredo navalis* and *Nototeredo norvagica* were the species with the widest distribution in Europe, but the *pedicellatus*-like *Lyrodus* lineages were also widely distributed in southern Europe. *Psiloteredo megotara* was reported occurring in the Atlantic coast of Europe but the number of occurrences reported for this species was small (Additional file 1). *Teredo bartschi* and *Teredothyra dominicensis* were found in European waters. The former was established in two sites (in the Atlantic and in the Mediterranean) and the latter occurred only in one site, in the eastern Mediterranean (Figure 2).

### Ecological requirements of teredinid species

**Figure 3:**
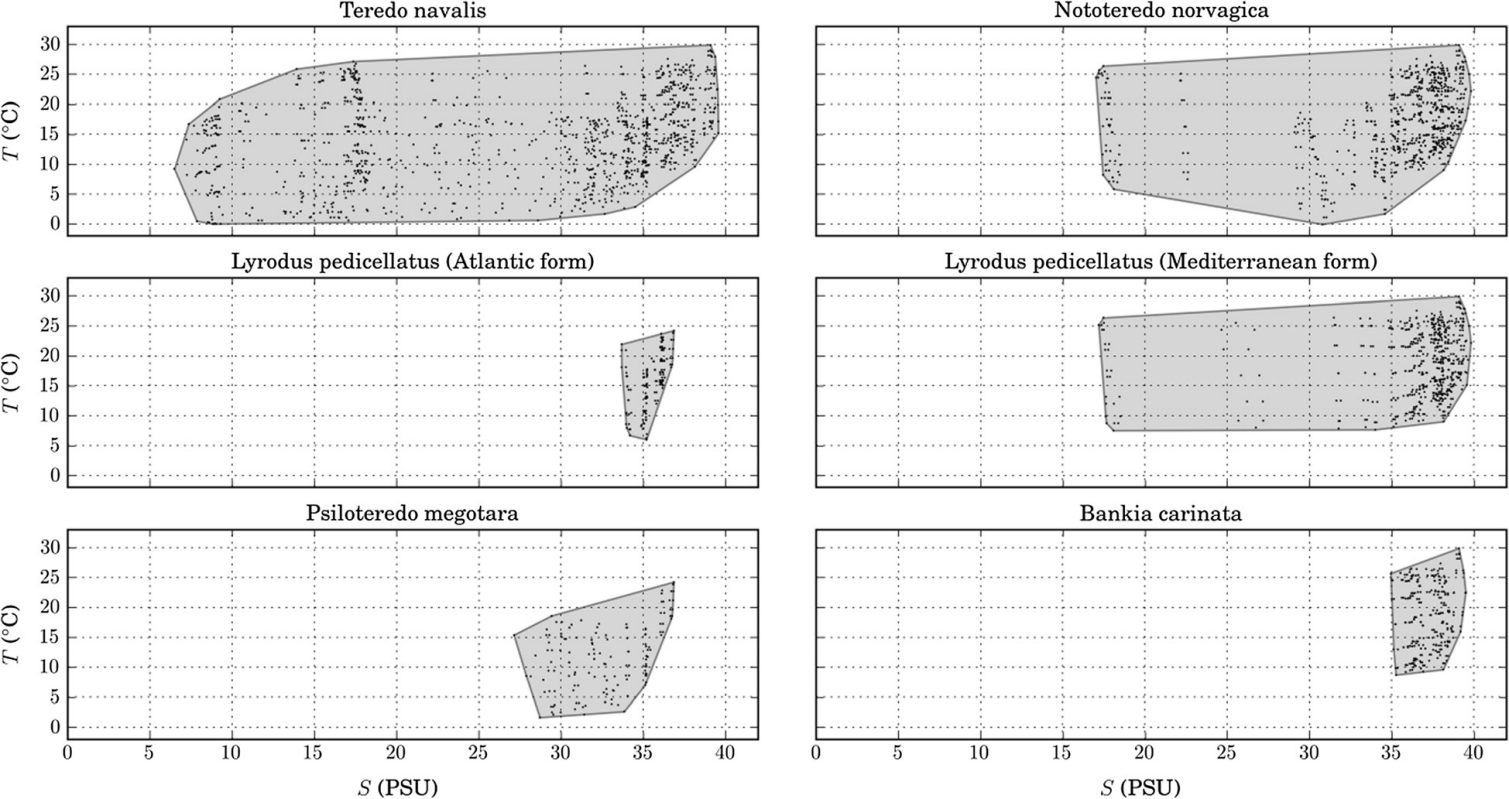
**Distribution of teredinid species in salinity-temperature space.** Distribution of teredinid species in salinity-temperature space. The minimum convex polygon encompassing all data points represent the climatic niche of each species.

Of all teredinids species occurring in European coastal waters, *Teredo navalis* has a particular wide tolerance for salinity and temperature. Its distribution in *S-T* space (Figure 3) showed that the species tolerates temperature and salinity ranges of 0–30°C and 7–39 PSU, respectively. Compared to *T. navalis*, *Nototeredo norvagica* also showed a wide temperature tolerance (2–30°C) but a narrower tolerance for salinity (17–39 PSU)*.*

The other species showed narrower temperature and salinity tolerances than the species mentioned above. *Psiloteredo megotara* showed a tolerance for temperature and salinity ranging from (1-25°C) and (27–37 PSU) respectively. *Bankia carinata* was the species with the highest requirements of temperature (9–30°C) and salinity (35–39 PSU). The temperature and salinity tolerance inferred in this study for the two lineages of *Lyrodus pedicellatus* might vary, as the exact limits of their distribution are not known (please see discussion below). *S-T* requirements were not determined for *Teredora malleolus*, *Teredo bartschi* and *Teredothyra dominicensis* because there are insufficient occurrences in Europe to permit their requirements to be characterised.

## Discussion

### Diversity of teredinids in European coastal waters

All species found on our field surveys were considered part of the local established fauna because they were able to grow to maturity and breed successfully in the areas where they were found [16], either in fixed wooden maritime structures or in our wooden collecting panels. We initially identified seven teredinid species, collected from field surveys, based on morphological characters according to the key of Turner [34]. However a recent study using molecular markers (mitochondrial and nuclear) revealed a genetic split between the Atlantic and eastern Mediterranean forms of the morphospecies *Lyrodus pedicellatus*[3]. Therefore we considered two lineages of *Lyrodus pedicellatus* occurring in European waters. However, a detailed phylogeographic study is needed to ascertain the geographic limits of each lineage. For this reason, we opted to show the distribution of the two lineages in the same figure (Figure 2 “*Lyrodus pedicellatus*”). Molecular evidence also showed that *N. norvagica* and *T. utriculus* are in fact a single species*, N. norvagica*, showing morphological plasticity [3].

*Teredora malleolus*, although not found in our field surveys, was found in timber structures submerged in Torbay [35], Weymouth, and Vorran Island, Scotland, UK [36], and in local wooden structures in Madeira and Azores, Portugal [15].Thus, this species was also considered established, as there were more than two occurrence records spread over time and space [37]. Therefore, there are nine established teredinid species in Europe.

In our field surveys specimens of *Teredo bartschi* recruited and matured in wooden panels exposed in the Atlantic (Olhão, Portugal) and in the Mediterranean (Mersin, Turkey), therefore this species was considered established in both areas [38]. *Teredothyra dominicensis* was found established in Kaş, Turkey [39].

In faunal compilations several other teredinid species have been reported to occur in Europe. These include *Bankia bipennata* (Turton, 1819), *Bankia fimbriatula* (Moll and Roch, 1931), *Teredo bipartita* (Jeffreys, 1860), synonym of *Lyrodus bipartita* (Jeffreys) [16], *Psiloteredo senegalensis* (Blainville, 1824), *Spathoteredo spatha* (Jeffreys, 1860) and *Teredothyra excavata* (Jeffreys, 1860). However, all specimens of these species were either found in driftwood carried by the Gulf Stream mainly to the British Isles [16] or in driftwood in the Mediterranean [15]. As far as we are aware, these species have never been reported as established in European coastal waters.

### Origin of teredinids established in European waters

Evidence gathered so far is still insufficient to answer the long standing question of the origin of the majority of teredinid species established in European waters. Although *Teredo navalis*, *Lyrodus pedicellatus*, *Nototeredo norvagica, Psiloteredo megotara and Teredora malleolus,* have their type locality in Europe, they should, along with *Bankia carinata*, be considered cryptogenic as there is no definite evidence either of their native or introduced status. However, it is possible that *N. norvagica* and *P. megotara* had their origin in Europe. *N. norvagica* has been reported mainly from Europe [6-8,15-17]. *P. megotara* also seems to occur mainly in Europe, particularly in western Norway, where it has been reported consistently in Trondheim Fjord since the 1920’s [40,41], although it was also sporadically reported occurring in the United States [16,42].

Additional evidence of the long-term occurrence in European waters of *Nototeredo norvagica* and *Psiloteredo megotara* is provided by the fossil record. Indeed fossil specimens, morphologically indistinguishable from, *N. norvagica* and *P. megotara* occurred in Europe during the upper Eocene [43]. However, it is not known whether populations of these species were able to survive, in glacial refugia, during the series of glacial cycles of the quaternary period, each of which temporarily erased many species from Europe [44]. Thus, further studies combining evidence from palaeontology and phylogeography, would be important to clarify the origin of teredinids in Europe.

*Teredo bartschi* was considered an alien species both in the Mediterranean and in the Atlantic [38]. Similarly, *Teredothyra dominicensis* was also considered to be an alien species in the Mediterranean and molecular studies revealed that the species originated from Caribbean populations [3,39].

#### Determining environmental requirement of coastal species using ‘bioclimatic envelopes’

Traditionally, the environmental requirements of coastal species have been determined in laboratory controlled conditions. However, the use of this method is difficult either when studying a large number of species or when studying species that are difficult to culture in laboratory conditions, for instance. In this study we showed that it is possible to use climatic envelopes to determine the environmental requirements of coastal species, using new oceanographic databases, such as BIO-ORACLE [20], which can resolve adequately coastal areas. The knowledge of these requirements is very useful to help explaining the biogeography of coastal species and determine their potential distribution (beyond the scope of this article) in areas that have not yet been surveyed.

### Biogeography of teredinids in the Atlantic and Baltic coasts of Europe

*T. navalis* has been the species of greatest economic concern in Europe because of its wide distribution and the threat it poses to wooden maritime structures [6,10,14,45-47]. Two main factors may explain the wide distribution of *T. navalis* in Europe. The first is that, according to our data, this species is both eurythermic and euryhaline, which corroborates previous findings by Roch [48] on its limits of temperature and the lower limit of salinity tolerated by the adults and larvae [17,24,49]. In laboratory experiments, Blum [24] observed *T. navalis* surviving at salinities as low as 2 PSU for 24 days, although the activity decreased abruptly below 9 PSU. Indeed, adult organisms have been reported surviving in areas that are either above or below their limits of temperature or salinity without detrimental effects. However *T. navalis* was never reported established in areas with such low salinities probably because this and other teredinid species require higher salinities for spawning and survival of the young [14,16,17].

The second factor is probably related to *Teredo navalis*´ life history. This species is a short-term larviparous [50]. This strategy provides protection to the larvae during the period of shell formation, the most vulnerable larval stage, while the free swimming period of 20 days, or even longer if wood is not available [17], allows the larvae to colonise new areas. Indeed, this life history strategy was considered the most effective to survive in patchy ephemeral habitats (wood) [28].

The recent distribution of *Teredo navalis* in the Baltic Sea suggests a range expansion into the eastern Baltic. Hoppe [51] observed that salinity conditions below 9 PSU prevented the establishment of *T. navalis* larvae in the Baltic Sea but recently this species has been observed occurring at salinities as low as 7 PSU (this study; Weigelt pers.com.). This might mean that *T. navalis* is adapting to lower salinity conditions, similarly to other species (e.g. gammarid amphipods) that colonised the Baltic during the “*Littorina* period”, when the salinity conditions were higher than they are at present. Indeed, many species that colonised the Baltic during this period were able to survive in the area, adapting to the ever decreasing salinity [52]. In addition *T. navalis* has little competition in the Baltic from other wood borers [47] and adult teredinids have few known predators [17], probably because of the protection provided by the wood.

**Figure 4:**
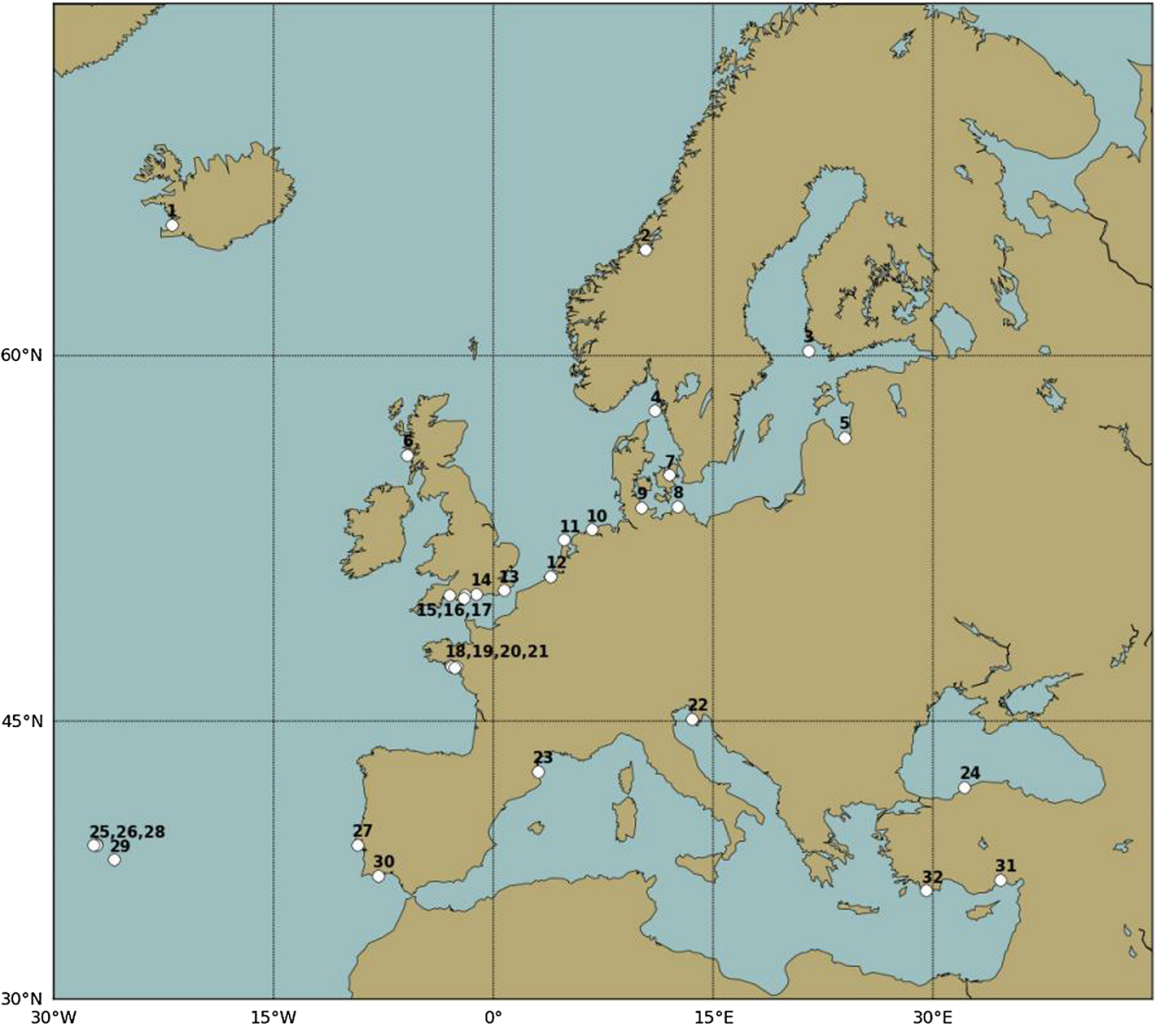
**Location of the 32 sites surveyed from 2001 to 2011.** Location of the 32 sites where teredinids were surveyed from 2001 to 2011.

While the distribution range of *T. navalis* in the Baltic seems to be expanding, recent occurrence data from the Atlantic coast of Europe seems to point towards a range contraction. This range contraction might be related to the dominance of the Atlantic form of *Lyrodus pedicellatus* in southwestern Europe, in areas such as the Tagus estuary, Lisbon, where *T. navalis* was dominant during the 1960’s and 1970’s [8]. The two species occur in sympatry in the majority of sites surveyed along the Atlantic coast of Europe (see Figure 4) up to the English Channel. In this sites *L. pedicellatus* was dominant. In the past, however, the occurrence of *L. pedicellatus* in the English Channel was reported as sporadic [50]. Recently *L. pedicellatus* was also reported occurring in the south of The Netherlands, North Sea [53]. These results seem to point to a range expansion of *L. pedicellatus* further north, outcompeting *T. navali*s.

The competitive advantage of the Atlantic form of *Lyrodus pedicellatus* is probably related to its life history strategy. The larvae of *L. pedicellatus*, a long-term brooder, are released as competent pediveligers. Given the presence of wood, they are able to settle immediately [50], whereas the larvae of *Teredo navalis* require a longer period to settle. However, according to our findings, the Atlantic form of *L. pedicellatus* seems to be moderately stenohaline, requiring salinities close to that of full-strength seawater to survive, which might limit its range expansion. Nevertheless, when conditions permit, this species may pose a great hazard to wooden maritime structures [54] and therefore should be closely monitored in Europe.

*Nototeredo norvagica* is also widely distributed in Europe (Figure 2), occurring in the majority of European waters, except in the Baltic Sea. This wide distribution is probably related to the wide range of temperature (eurythermic) and salinity (moderately euryhaline) tolerated by this species. The salinity in the Baltic Sea may be too low, preventing the occurrence of *N. norvagica* in the area. Although *N. norvagica* is widely distributed in Europe, it does not seem to pose great hazard to coastal wooden maritime structures. This might be related to the life history strategy of this species. Indeed, *N. norvagica* seems to avoid competition with other teredinids by breeding at a later stage (autumn or early winter, as opposed to spring or early summer). It also seems to settle preferentially at greater depths [16,17,55], which may pose a threat for maritime archaeological structures located at these greater depths.

*Psiloteredo megotara* is another species that occurs in the Atlantic coast of Europe, which similarly to *Nototeredo norvagica*, does not seem to pose a great threat to coastal wooden structures. This species has been reported to occur from Norway to Madeira, Portugal (Figure 2). However, it is possible that the similarity between the pallets of *P. megotara* and *N. norvagica* has produced some erroneous identifications in the former species for instance in southern Europe [54]. Indeed, this species seems to be better adapted to survive in colder waters, where it grows continuously even at temperatures as low as 5°C, attaining great lengths, whereas *T. navalis* ceases growth at such low temperatures [47].

### Biogeography of teredinids in the Mediterranean and Black Seas

The occurrence of a *pedicellatus*-like *Lyrodus* in the Black Sea [9] seems to point towards a recent range expansion of the Mediterranean form of *L. pedicellatus*, similarly to what was observed in the Atlantic form of *L. pedicellatus*. Morphologically, specimens of *L. pedicellatus* from the Mediterranean and the Black Sea are identical (LB, personal observation), but sequence data are required to determine whether they belong to a single species or not. However, the proximity of the two water basins makes it probable that a range expansion has occurred. The Mediterranean form of *Lyrodus pedicellatus* occurs in sympatry with other teredinid species such as *Teredo navalis*, *Bankia carinata and Nototeredo norvagica*. In our long-term field survey (2002/03) the former species was dominant in the two Mediterranean sites surveyed (Rovinj, Croatia and Mersin, Southern Turkey). However in a later study (2007), the dominant species in Mersin was *Teredo navalis*[6]. *Teredo navalis* and *Lyrodus pedicellatus* have a very high activity in the Mediterranean posing a great hazard to wooden maritime structures [54] and they should, therefore, be monitored, in particular in areas where wood is extensively used in maritime construction.

The other species reported upon in this study seem to have more restricted distributions. *Bankia carinata* is a warm water species occurring worldwide [36] but in European waters has only been found in the Mediterranean. However, the inferred limits of temperature for this species show a relatively wide temperature tolerance (9-30°C). Thus its narrow salinity tolerance may be limiting the distribution of this species. The results of this study suggest that the species is stenohaline with a preference for hyperhaline conditions (35–39 PSU), surviving even at salinities of over 40 PSU, in the Gulf of Aqaba [29]. *Bankia carinata* and *Teredo bartschi* were found to occur in the Red Sea [29]. Therefore, it is possible that both species might have entered the Mediterranean via the Suez Canal (lessepsian migrants), although in the case of *B. carinata*, at a much earlier stage as records of its presence in the European coast of the Mediterranean date back to the beginning of the 20th Century [36]. However, molecular data (mitochondrial and nuclear markers) are necessary to test this hypothesis. Indeed molecular data revealed that *Teredothyra dominicensis*, the most recent teredinid invader into the Mediterranean, originated from Caribbean populations [3,39].

## Conclusions

The data obtained from field surveys, coupled with a comprehensive review of the literature, made it possible to ascertain the diversity of established teredinid species in Europe, and also their past and recent distribution. The use of molecular markers [3] improved the ‘taxonomic resolution’ in the *Lyrodus pedicellatus* complex of species and in *Nototeredo norvagica*, which was important to study the biogeography of teredinid species in European waters.

We showed that it is possible to use the new high-resolution oceanographic datasets to determine the environmental requirements of coastal benthic organisms, when occurrence data is available, using climatic envelopes. This method can speed up the acquisition of environmental requirement data of coastal species, at a fraction of the cost, when compared to laboratory controlled experiments.

By comparing the past occurrence of teredinids (before 2000) with recent occurrence data (after 2000), we were able to observe trends on species range extension or contraction, and competition. These trends also confirmed the importance of temperature and salinity on the distribution of teredinid species. Indeed the two species with wider distribution in Europe, *Teredo navalis* and *Nototeredo norvagica*, are also the ones with the widest temperature and salinity tolerance, in spite of having different life history strategies (short-term larviparous and oviparous, respectively). On the other hand, the type of life history strategy of the Atlantic form of *L. pedicellatus* (long-term larviparous) seems to give it a competitive advantage over *Teredo navalis* (short-term larviparous). In the case of the Mediterranean form of *L. pedicellatus* it is not clear whether this species also has a competitive advantage over *T. navalis*, because nothing is known about the life history strategy of this putative new species.

Of all species found established in European coastal waters, *Teredo navalis* and the two lineages of *Lyrodus pedicellatus* are the ones of greatest concern due to their wide distribution and destructive capacity [54] and they should, therefore, be monitored further. The two alien species, *Teredo bartschi* and *Teredothryra dominicensis*, should also be monitored to assess whether they are spreading in European waters. Recently observed trends, show that the changing climate has a profound influence on the range expansion and contraction of species [21]. Therefore we can anticipate that given the envelopes of tolerance for teredinid species, some will change their range, expanding or contracting, depending on their tolerances.

## Methods

### Occurrence data

To collect data on the diversity and distribution of teredinid species in European coastal waters, we carried out field surveys at 32 different sites (Table 1; Figure 4), between 2001 and 2011, some of which were surveyed more than once (e.g. Olhão, Portugal; Mersin, Turkey). One of these was a long-term survey conducted at 15 sites, which were selected to represent the range of seawater temperature and salinity conditions in Europe. The sites ranged from sub-tropical waters in Mersin, Turkey to colder waters in Reykjavik, Iceland, encompassing diverse salinity conditions, from hyperhaline in the eastern Mediterranean to hypohaline in the Baltic.

In the long-term survey, six replicate Scots pine (*Pinus sylvestris L.*) panels, with dimensions 20x10x2 cm, were deployed at 2–3 m depth at each site in May 2002 and used as baits for teredinid settlement. Scots pine was chosen because it is a non-durable timber species used in standard tests of wood durability as comparator [56]. The exposure of the panels (May 2002) was timed to coincide with the period of higher larval settlement for most teredinid species known to occur in Europe and also to avoid the settlement of fouling organisms on the wood surface, which creates a physical barrier to settlement of teredinid larvae. After one year of exposure, the panels were retrieved and split up to extract the organisms. Additional data on the occurrence of teredinid species were obtained by collecting wood from fixed structures in opportunistic surveys (e.g. Swanage Pier, Rye Admiralty Jetty, groynes at Bournemouth beach, UK) and also from 3 sites, during a collaboration in the project MoSS (Monitoring, Safeguarding and Visualizing North European Shipwreck sites) [57]. Morphological identification of specimens was based on the diagnostic characters of the pallets, using the keys of Turner [34] and plates in Turner [16]. The identity of the majority of species was confirmed using mitochondrial, cytochrome *c* oxidase subunit I (COI-5P), and nuclear 18S rDNA sequence data [1,3,39].

**Table 1:** Sites surveyed, geographic coordinates (decimal degrees), time and duration of surveys and type of structure from which teredinids where collected from

**Sites surveyed**	**Coordinates**	**Year(s) and duration**	**Type of structure**
**1 Reykjavik-Iceland**	64.15; -21.90	2002/2003 (1 year)	Wooden panels
**2 Trondheim-Norway**	63.41; 10.41	2002/2003 (1 year)	Wooden panels
**3 Island of Jurmo-Finland**	60.15; 21.57	2002 (1 year)	Wooden panels at shipwreck site
**4 Kristineberg Marine Station-Sweden**	58.03; 11.05	2002/2003 (1 year)	Wooden panels
**5 Riga-Latvia**	57.06; 24.03	2002/2003 (1 year)	Wooden panels
**6 Oban-Scotland**	56.41; -5.80	2002/2003 (1 year)	Wooden panels
**7 Roskilde-Denmark**	55.63; 12.08	2002/2003 (1 year)	Wooden panels
**8 Mouth of River Prerowstorm-Germany**	54.41; 12.61	2002 (1 year)	Wooden panels at shipwreck site
**9 Kiel-Germany**	54.36; 10.15	2002/2003 (1 year)	Wooden panels
**10 Haren-Netherlands**	53.48; 6.76	2002/2003 (1 year)	Wooden panels
**11 Near Texel-Netherlands**	53.06; 4.90	2002 (1 year)	Wooden panels at shipwreck site
**12 Yerseke-Netherlands**	51.53; 3.98	2002/2003 (1 year)	Collecting panels
**13 Rye-England**	50.93; 0.76	2001 (1 day)	Old wooden piles
**14 Portsmouth-England**	50.79; -1.02	2002/2003; 2003/2004 (2 years)	Wooden panels
**15 Lyme Regis-England**	50.72; -2.93	2004 (1 day)	Samples of local wooden structures
**16 Bournemouth-England**	50.71; -1.87	2003 (1 day)	Old wooden piles
**17 Swanage-England**	50.60; -1.95	2001 (1 day)	Samples from the old pier
**18 Toulindac-France**	47.60; -2.87	2008 (1 day)	Wooden structures
**19 Golfe du Morbihan-France**	47.56; -2.79	2009 (1 day)	Wooden structures
**20 Berder-France**	47.55; -2.48	2008 (1 day)	Wooden structures
**21 Penerf-France**	47.50; -2.61	2009 (1 day)	Wooden structures
**22 Rovinj-Croatia**	45.08; 13.63	2002/2003 (1 year)	Wooden panels
**23 Banyuls-sur-mer-France**	42.48; 3.12	2009 (1 day)	Wooden structures
**24 Bartin- Turkey**	41.68; 32.22	2002/2003 (1 year)	Wooden panels
**25 Praia da Vitória-Azores**	38.71; -27.04	2002/2003 (1 year)	Wooden panels
**26 Porto Martins-Azores**	38.68; -27.05	2001 (1 day)	Wooden structures
**27 Lisbon- Portugal**	38.67; -9.20	1990/2006 (16 years)	Old wooden piles
**28 Angra do Heroísmo-Azores**	38.65; -27.21	2001 (1 day)	Wooden structures
**29 Mosteiros-Azores**	37.88; -25.80	2011 (1 week)	Wooden structure?
**30 Olhão-Portugal**	37.00; -7.79	2002/2003; 2003/2004 (2 years)	Wooden panels
**31 Mersin-Turkey**	36.80; 34.64	2002/2003; 2006/2007 (2 years)	Wooden panels
**32 Kaș-Turkey**	36.19; 29.63	2010 (1 week)	Samples from the shipwreck

In addition to the field surveys, we compiled records obtained from a comprehensive survey of the primary literature dating back to the 1900 (e.g. [15]), specific works on teredinids (e.g. [16,17]), faunal compilations (e.g. [18]), unpublished reports and online databases (e.g. [36,53,58,59]). Occurrence data obtained from the field surveys and from the literature were compiled in a database containing, the locality name, geographic coordinates and year of occurrence (Additional file 1), and then mapped as: a) past distributions-occurrences reported before 2000; b) recent distribution- data from the field surveys and from published reports since 2000. Synonyms were updated and chresonyms reviewed using the publications of [16,19] to present the most accurate taxonomic nomenclature currently accepted for the Teredinidae. Special care was taken to eliminate dubious occurrences, for instance when identifications were based on shells, or specimens were found in driftwood only.

### Data on temperature and salinity

Our collaborators provided locally measured temperature and salinity data on 13 sites surveyed in Europe. However we needed a global dataset to provide environmental data for the occurrences obtained from the other surveys and from the literature review. Therefore, we used a hybrid dataset primarily based on BIO-ORACLE [20], which provides global data on sea surface temperature (SST) and sea surface salinity (SSS), amongst other parameters, gridded with a spatial resolution of 5 arcmin (ca. 9.2 km). In BIO-ORACLE, SST data (minimum value, mean value and range) were compiled from satellite imagery and provided as a climatology, *i.e*. averaged over 20 years [20]. We parameterized the monthly variability by a phase-shifted cosine function with a period of 1 year, such that the minimum temperature was achieved in January and the maximum in July. Salinity data in the BIO-ORACLE dataset were compiled from *in situ* observations, interpolated to a grid and provided as a yearly average only [20]. To add information on the monthly variability of salinity, we superposed to each wet grid cell in the BIO-ORACLE dataset, the monthly long-term salinity anomaly from the nearest wet grid cell in the dataset, provided by the Research Data Archive (RDA). The RDA dataset contains global monthly averaged values of temperature and salinity for the period 1948–2012 [60], but at a coarse resolution of about 100 × 100 km.

The resolution of coastal areas in the North and Baltic Seas in the BIO-ORACLE/RDA compiled dataset was further improved by including the output from a general circulation model, available from the Coastal Observation System for North and Arctic Seas (COSYNA), a pre-operational coastal observatory [27]. Since mid-2010, the model (based on the General Estuarine Transport Model) runs daily using realistic atmospheric forcing and riverine input, on a curvilinear grid with a resolution of about 5 × 5 km [61]. The resulting hybrid dataset used in this work is a synthesis of the COSYNA model output for the North Sea and Baltic Sea, and the BIO-ORACLE/RDA dataset everywhere else.

**Figure 5:**
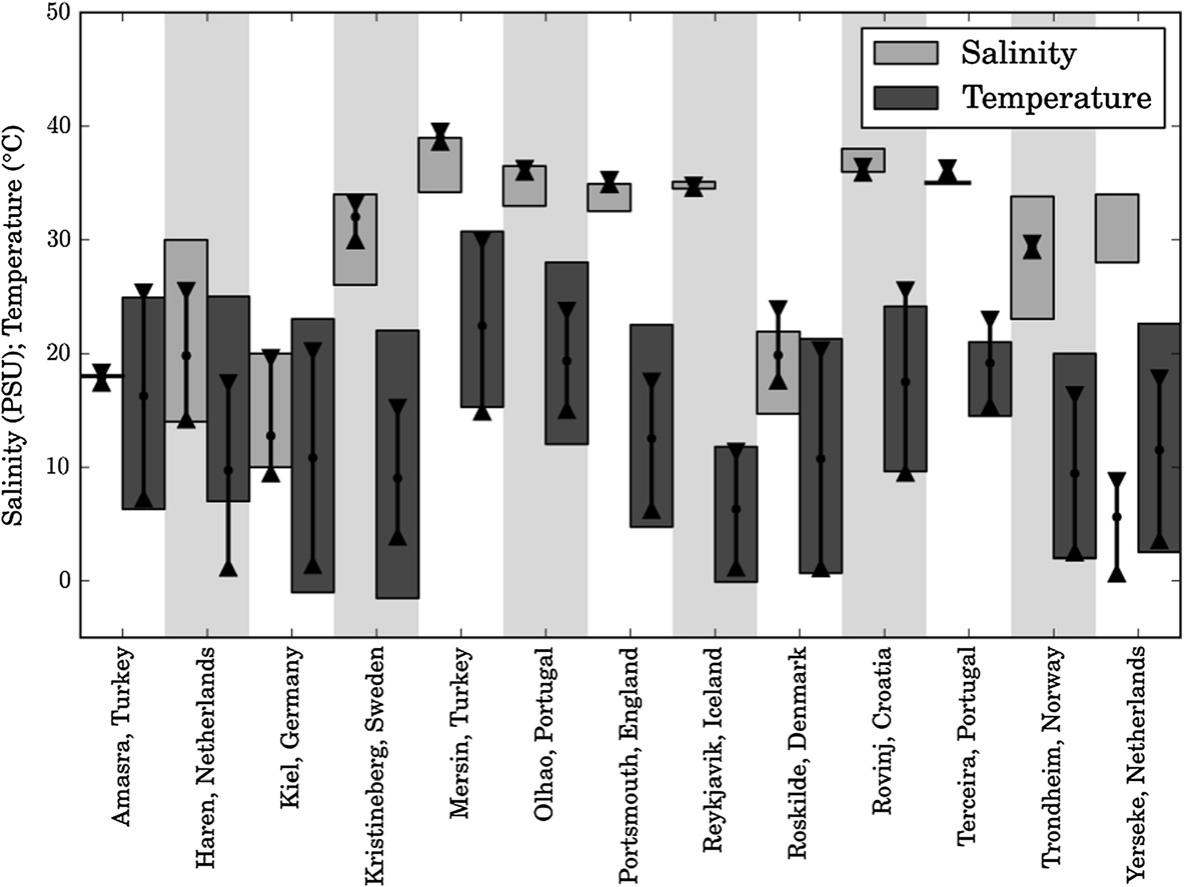
**Comparison of the *S-T *hybrid dataset with measured data.** Comparison of the hybrid dataset values for temperature and salinity with measured data from 13 sites where panels were deployed. The measured ranges are represented by the shaded vertical boxes, except for the salinity reported for Terceira and Amasra, for which we only have the mean values (indicated by horizontal lines). The ranges from minimum (triangles pointing up) to maximum (triangles pointing down) values obtained from the hybrid dataset are indicated by vertical lines, with dots indicating the mean values.

To assess the accuracy of the hybrid dataset, we compared the data with data obtained from measurements at 13 sites where panels were deployed (Figure 5). The spatial resolution of the hybrid dataset (5 km) was sufficient to resolve both temperature and salinity in coastal waters and was representative for most sites for which measured data were available. Nevertheless, discrepancies were still observed between the salinity dataset and measured local conditions in estuarine areas. This is to be expected as the salinity conditions in estuaries can be highly variable and require even higher resolution models. In Yerseke, Netherlands, a tidal area, the hybrid dataset failed to represent the salinity conditions of the partially dammed sea arm and, therefore, this datum was excluded from the dataset.

### Temperature and salinity tolerated by the European teredinid species

To understand the climatic conditions suitable for the survival of teredinid species, a climatic envelope was defined in salinity-temperature (*S-T*) space. To construct the habitable region in *S-T* space for a given species, it was assumed that the species tolerated all salinity and temperature combinations at the sites where they were reported. We, therefore, averaged the monthly values of salinity and temperature at each site. Thus, for a given year each site yields 12 *S-T* data points. The effects due to seasonal variation were accounted for, but short-term variations (weekly or daily) were ignored. The climatic envelope was then defined as the area enclosed by the minimum convex polygon encompassing all data points.

## Competing interests

The authors declare that they have no competing interests.

## Authors’ contributions

LB conceived the study and its design, collected and analysed the data, and drafted the manuscript. LM developed software to determine the climatic envelopes and helped producing the maps of species occurrence. ÍS analysed the data and helped producing the maps of species occurrence. SC also conceived the study and its design, and coordinated the study. All authors read and approved the final manuscript.

## Supplementary Material

Additional file 1 Distribution of bivalve wood borers (Teredinidae) in European coastal waters. Click here for file
