# Supplementary material for: Diversity, environmental requirements, and biogeography of bivalve wood-borers (Teredinidae) in European coastal waters
Source: Front Zool. 2014 Feb 13;11:13. doi: 10.1186/1742-9994-11-13 (PMC3925441; doi:10.1186/1742-9994-11-13)
Supplement: Additional file 1 — Distribution of bivalve wood borers (Teredinidae) in European coastal waters. [file 1742-9994-11-13-S1.docx]

Table 2 Distribution of bivalve wood borers (Teredinidae) in European coastal waters.

| Species/ authority | Location | Coordinates | | | Source |
| --- | --- | --- | --- | --- | --- |
| *Teredo navalis* Linnaeus, 1758 | North of Trondheim-Norway | 63.88; 9.83 | | | Sneli, pers. com. (compiled by Brattegard, 2001) |
|  | Bergen-Norway | | 60.39; 5.32 | Nair, 1962; Santhakumaran and Sneli, 1984 | |
|  | Drobak-norway | | 59.66; 10.62 | Jones et al., 1972 | |
|  | Kristineberg Marine Biological Station-Sweden | | 58.03; 11.05 | Norman, 1977; this study (May 2002 to May 2003) | |
|  | Gothenburg-Sweden | | 57.67; 11.86 | Petterson, 1925 in Clapp and Kenk, 1963 | |
|  | Hirtshals harbour-Denmark | | 56.98; 10.30 | kramp, 1937 in Clapp and Kenk, 1963 | |
|  | Kattegat | | 56.95;11.30 | Kristensen, 1969 | |
|  | Kyholm-Denmark | | 55.93; 10.68 | Kristensen, 1969 | |
|  | Copenhagen-Denmark | | 55.70; 12.65 | Kristensen, 1969 | |
|  | Roskilde-Denmark | | 55.63; 12.08 | This study (May 2002 to May 2003) | |
|  | Flensburg Bay-Germany | | 54.78;9.43 | Hoppe, 2002 | |
|  | Gjedser-Denmark | | 54.57; 11.92 | Troshcel 1913a in Sordyl et al., 1998 | |
|  | Hiddensee-Germany | | 54.54; 13.01 | Sordyl et al., 1998 | |
|  | Island of Rügen-Germany | | 54.44; 13.57 | Sordyl et al., 1998 | |
|  | Zingst-Germany | | 54.44; 12.68 | Becker, 1938; Sordyl et al., 1998 | |
|  | Ahrenshoop-Germany | | 54.38; 12.41 | Sordyl et al., 1998 | |
|  | Foresterei Timbrook-Germany | | 54.36;10.11 | This study (2011) | |
|  | Kiel-Germany | | 54.36; 10.15 | Troschel, 1913 in Clapp and Kenk, 1963; Schutz, 1961 in Kristensen, 1969; Jones et al., 1972; this study (2002, 2003) | |
|  | Wustrow- Germany | | 54.35; 12.38 | Sordyl et al., 1998 | |
|  | Markgrafenheide-Germany | | 54.18; 12.10 | Sordyl et al., 1998 | |
|  | Warnemünde-Germany | | 54.18; 12.07 | Eckstein, 1928 in Clapp and Kenk, 1963; Sordyl et al., 1998; | |
|  | Helgoland-Germany | | 54.17; 7.89 | Troshcel, 1913 in Clapp and Kenk, 1963; Moll, 1940; Caspers, 1941; | |
|  | Kühlungsborn-Germany | | 54.15; 11.76 | Sordyl et al., 1998 | |
|  | Heiligendamm-Germany | | 54.15; 11.74 | Bavendammm, 1948 | |
|  |  | |  |  | |

Table 2 (continued) Distribution of bivalve wood borers (Teredinidae) in European coastal waters.

| Species/ authority | Location | | Coordinates | Source |
| --- | --- | --- | --- | --- |
| *Teredo navalis* Linnaeus, 1758 | Büsum-Germany | | 54.12; 8.86 | Jaeckel, 1928; Moll, 1940 |
|  | Cuxhaven-Germany | 53.88; 8.72 | | Windolf, 1936; Becker, 1938; Tuente et al., 2002 |
|  | Wangerooge Island-Germany | | 53.79; 7.9 | Schwarz, 1932 |
|  | East Frisian Islands-Germany | | 53.73; 7.41 | Leege, 1939 |
|  | Borkum -Germany | | 53.57; 6.66 | Roch, 1937 |
|  | Bremerhaven-Germany | | 53.54; 8.58 | Tuente et al., 2002 |
|  | Wilhelmshaven-Germany | | 53.51; 8.12 | Troschel, 1913; Moll and Roch, 1929; Becker, 1938; Tuente et al., 2002 |
|  | Haren-Netherlands | | 53.48; 6.76 | This study (May 2002 to May 2003) |
|  | Liverpool-England | | 53.45; -3.03 | Newell, 1920 |
|  | Texel-Netherlands | | 53.09; 4.80 | Palma, 2005 |
|  | Scheveningen-Netherlands | | 52.09; 4.25 | Anon, 1940 in Clapp and Kenk, 1963 |
|  | Rotterdam Harbour-Netherlands | | 51.94; 4.07 | Paalvast and Velde, 2011 |
|  | Yerseke-Netherlands | | 51.53; 3.98 | This study (May 2002 to May 2003) |
|  | Southhead, River Thames-England | | 51.52; 0.72 | Hall and Saunders, 1967 |
|  | Ostende-Belgium | | 51.22; 2.88 | Leloup and Miller, 1940 |
|  | Nieuport-Belgium | | 51.15; 2.72 | Loppens, 1905 in Clapp and Kenk, 1963 |
|  | Dover-England | | 51.11; 1.31 | Hall and Saunders, 1967 |
|  | Plymouth-England | | 50.86; -4.13 | Hall and Saunders, 1967 |
|  | Shoreham-England | | 50.82; -0.25 | Hall and Saunders, 1967 |
|  | Devonport-England | | 50.37; -4.18 | Hayes, 1922 |
|  | Plymouth-England | | 50.36; -4.15 | Scott, 1910 |

Table 2 (continued) Distribution of bivalve wood borers (Teredinidae) in European coastal waters.

| Species/ authority | Location | Coordinates | | Source |
| --- | --- | --- | --- | --- |
| *Teredo navalis* Linnaeus, 1758 | Berder-France | 47.58;-2.88 | | This study (2008) |
|  | Golfe du Morbihan-France | | 47.56; -2.79 | This study (2009) |
|  | Baie de Villaine-France | | 47.54;-3.06 | This study (2008) |
|  | Muggia-Italy | | 45.60;13.75 | Roch, 1940 |
|  | Kertsch-Ukraine | | 45.35; 36.48 | Roch, 1940 |
|  | Venice-Italy | | 45.35; 12.38 | Roch, 1940; Cormio, 1947 in Clapp and Kenk, 1963 |
|  | Toulindac-France | | 45.34;-2.52 | This study (2008) |
|  | Rijeka-Croatia | | 45.32; 14.44 | Roch, 1940 |
|  | Eupatoria-Ukraine | | 45.18; 33.37 | Roch, 1940 |
|  | Canal di Leme-Italy | | 45.13; 13.60 | Roch, 1940 |
|  | Rovinj-Croatia | | 45.08;13.63 | Roch, 1940; this study (May 2002 to May 2003) |
|  | Feodosyia-Ukraine | | 45.02; 35.39 | Roch, 1940 |
|  | Anapa- Russia | | 44.89; 37.30 | Roch, 1940 |
|  | Pula-Croatia | | 44.86; 13.84 | Roch, 1940 |
|  | Noworossijsk-Russia | | 44.72; 37.89 | Roch, 1940 |
|  | Jalta-Ukraine | | 44.49; 34.16 | Roch, 1940 |
|  | Ravenna-Italy | | 44.48; 12.29 | Roch, 1940 |
|  | Constanta Romania | | 44.15; 28.66 | Antipa, 1941 in Clapp and Kenk, 1963 |
|  | Turzla-Romania | | 44.03; 28.65 | Antipa, 1941 in Clapp and Kenk, 1963 |
|  | Ancona-Italy | | 43.62; 13.50 | Roch, 1940 |
|  | Marseille-France | | 43.29; 5.36 | Henry, 1909 |
|  | Varna-Bulgaria | | 43.18; 27.92 | Roch, 1940 |
|  | Suchum-Georgia | | 42.99;40.99 | Roch, 1940 |
|  | San Benedetto del Tronto-Italy | | 42.95; 13.89 | Roch, 1940 |
|  | Batum-Georgia | | 42.64; 41.65 | Roch, 1940 |

Table 2 (continued) Distribution of bivalve wood borers (Teredinidae) in European coastal waters.

| Species/ authority | Location | Coordinates | Source |
| --- | --- | --- | --- |
| *Teredo navalis* Linnaeus, 1758 | Banyuls-sur-Mer-France | 42.47; 3.11 | This study (2008) |
|  | Pescara-Italy | 42.46; 14.23 | Roch, 1940 |
|  | Amasra-Turkey | 41.68; 32.22 | This study (May 2002 to May 2003; 2010) |
|  | Eregli-Turkey | 41.26; 31.41 | Sen et al., 2010 |
|  | Bari-Italy | 41.11; 16.87 | Roch, 1940 |
|  | Tarragona-Spain | 41.10; 1.25 | Roch, 1940 |
|  | Istanbul-Turkey | 41.01; 28.98 | Roch, 1940 |
|  | Trabazon-Turkey | 41.00; 39.73 | Sen et al., 2010 |
|  | Naples-Italy | 40.84; 14.26 | Roch, 1940 |
|  | Thessaloniki-Greece | 40.63; 22.93 | Roch, 1940 |
|  | Bandirma-Turkey | 40.35 27.96 | Sen et al., 2010 |
|  | Daphni-Greece | 39.93; 23.58 | Moll, 1940 |
|  | Valencia-Spain | 38.99; -0.14 | Benito Martinez, 1952 |
|  | Lisbon-Portugal | 38.67; -9.20 | Jones et al., 1972 |
|  | Gulf of Patras-Greece | 38.33; 21.65 | Nieder, 1883 in Clapp and Kenk, 1963 |
|  | Cesme-Turkey | 38.32; 26.29 | Sen et al., 2010 |
|  | Messina-Italy | 38.19; 15.26 | Roch, 1940 |
|  | Mersin-Turkey | 36.80; 34.64 | This study (May 2002 to May 2003; 2006/ 2007) |
|  | Kos-Greece | 36.75; 27.11 | Roch, 1940 |
|  | Malaga-Spain | 36.71; -4.42 | Roch, 1940 |
|  | Iskenderun-Turkey | 36.59; 36.17 | Sen et al., 2010 |
|  | Finike-Turkey | 36.29; 30.14 | Sen et al., 2010 |
| *Lyrodus pedicellatus (Quatrefages, 1849)* | Plymouth-England | 50.86; -4.13 | Lebour, 1938 |
|  | Hamble Estuary-England | 50.84;-1.31 | Coughlan, 1977 |
|  | Portsmouth-England | 50.79; -1.02 | This study (May 2002 to May 2003; 2004; 2005) |

Table 2 (continued) Distribution of bivalve wood borers (Teredinidae) in European coastal waters.

| Species/ authority | Location | Coordinates | Source |
| --- | --- | --- | --- |
| *Lyrodus pedicellatus (Quatrefages, 1849)* | Newton Ferrers-England | 50.30; -4.07 | Hall and Saunders, 1967 |
|  | Golfe du Morbihan-France | 47.56; -2.79 | This study (2009) |
|  | Sistiana-Italy | 45.76; 13.62 | Roch, 1940 |
|  | Gulf of Trieste-Italy | 45.64; 13.75 | Roch, 1940 |
|  | Muggia-Italy | 45.60;13.75 | Roch, 1940 |
|  | Venice-Italy | 45.41; 12.35 | Cormio, 1947 in Clapp and Kenk, 1963 |
|  | Rijeka-Croatia | 45.32; 14.44 | Roch, 1940 |
|  | Parec- Croatia | 45.22; 13.59 | Roch, 1940 |
|  | Chioggia-Italy | 45.22; 12.39 | Roch, 1940 |
|  | Omišalj-Croatia | 45.20; 14.55 | Roch, 1940 |
|  | Canal di Leme-Italy | 45.13; 13.60 | Roch, 1940 |
|  | Rovinj-Croatia | 45.08; 13.63 | Roch, 1940; Cormio, 1947 in Clapp and Kenk, 1963; this study (May 2002 to May 2003) |
|  | Brijoni Islands-Croatia | 44.91; 13.76 | Cormio, 1947 in Clapp and Kenk, 1963 |
|  | Lussinpiccolo-Croatia | 44.53; 14.46 | Roch, 1940 |
|  | Marina di Ravenna-Italy | 44.48; 12.29 | Roch, 1940 |
|  | Zadar-Croatia | 44.11; 15.22 | Roch, 1940 |
|  | La Spezia-Italy | 44.09; 9.82 | Roch, 1940 |
|  | Ancona-Italy | 43.62; 13.50 | Roch, 1940 |
|  | Split-Croatia | 43.50; 16.44 | Roch, 1940 |
|  | Marseille-France | 43.29; 5.36 | Roch, 1940 |
|  | Komiža, Croatia | 43.04; 16.08 | Roch, 1940 |
|  | San Benedetto del Tronto-Italy | 42.95; 13.89 | Roch, 1940 |
|  | Lastovo-Croatia | 42.73; 16.88 | Roch, 1940 |
|  | Pescara-Italy | 42.46; 14.23 | Roch, 1940 |
|  | San Domino-Italy | 42.11; 15.49 | Roch, 1940 |

Table 2 (continued) Distribution of bivalve wood borers (Teredinidae) in European coastal waters.

| Species/ authority | Location | Coordinates | Source |
| --- | --- | --- | --- |
| *Lyrodus pedicellatus (Quatrefages, 1849)* | Vieste-Italy | 41.88; 16.17 | Roch, 1940 |
|  | Barcelona-Spain | 41.41; 2.23 | Giribet and Peñas, 1997 |
|  | Eregli-Turkey | 41.26; 31.41 | Sen et al., 2010 |
|  | Adriatic Sea | 41.23; 16.86 | Cormio, 1947 in Clapp and Kenk, 1963 |
|  | Bari-Italy | 41.11; 16.87 | Roch, 1940 |
|  | Tarragona-Spain | 41.10; 1.25 | Roch, 1940 |
|  | Trabazon-Turkey | 41.00; 39.73 | Sen et al., 2010 |
|  | Naples-Italy | 40.84; 14.26 | Roch, 1940 |
|  | Thessaloniki-Greece | 40.63; 22.93 | Roch, 1940 |
|  | Alghero, Sardinia-Italy | 40.56; 8.31 | Roch, 1940 |
|  | Taranto-Italy | 40.49; 17.31 | Roch, 1940 |
|  | Bandirma-Turkey | 40.35 27.96 | Sen et al., 2010 |
|  | Galliopoli-Italy | 40.05; 17.98 | Roch, 1940 |
|  | Volo-Greece | 39.36; 22.94 | Roch, 1940 |
|  | Praia da Vitória-Portugal | 38.71; -27.04 | This study (May 2002 to May 2003) |
|  | Lisbon-Portugal | 38.67; -9.20 | This study (2007) |
|  | Angra do Heroísmo-Portugal | 38.65; -27-21 | Roch, 1940 |
|  | Cesme-Turkey | 38.32; 26.29 | Sen et al., 2010 |
|  | Messina-Italy | 38.19; 15.26 | Roch, 1940; Jones et al., 1972 |
|  | Piräus-Greece | 37.94; 23.62 | Roch, 1940 |
|  | Mosteiros-Portugal | 37.90; -25.81 | This study (2011) |
|  | Olhão -Portugal | 37.00; -7.79 | Brown, 1998; Praël, 2003; this study ( May 2002 to May 2003) |
|  | Mersin Bay-Turkey | 36.80; 34.64 | Bobat, 1995; this study (May 2002 to May 2003; 2007) |
|  | Kos-Greece | 36.75; 27.11 | Roch, 1940 |
|  | Malaga-Spain | 36.71; -4.42 | Roch, 1940 |

Table 2 (Continued) Distribution of *Teredo navalis* in European coastal waters.

| Species/ authority | Location | Coordinates | Source |
| --- | --- | --- | --- |
| *Lyrodus pedicellatus (Quatrefages, 1849)* | Iskenderun-Turkey | 36.59; 36.17 | Sen et al., 2010 |
|  | Rhodes-Greece | 36.44; 28.23 | Roch, 1940 |
|  | Finike-Turkey | 36.29; 30.14 | Sen et al., 2010 |
|  | La Valleta-Malta | 35.98; 14.51 | Roch, 1940 |
|  | Larnaca-Cyprus | 34.79; 33.56 | Roch, 1940 |
|  | Funchal- Madeira | 32.64;-16.91 | Roch, 1940 |
| *Psiloteredo megotara* (Hanley, 1848) | Trondheim-Norway | 63.41; 10.41 | Dons, 1949; Santhakumaran and Sneli, 1984; this study (May 2002 to May 2003) |
|  | Bergen-Norway | 60.38; 5.30 | Nair, 1962 |
|  | Lerwick-Scotland | 60.15;-1.14 | Jeffreys, 1860 in Turner, 1966 |
|  | Kristineberg-Sweden | 58.03; 11.05 | Norman, 1977; Henningsson and Norman, 1980 |
|  | Harbour of Hirtshals-Denmark | 57.59; 9.97 | Kramp, 1937 in Clapp and Kenk, 1963 |
|  | Frederikshavn-Denmark | 57.43; 10.55 | Kramp, 1944 in Kristensen, 1969 |
|  | Borkum-Germany | 53.57; 6.66 | Roch, 1937 |
|  | Scheveningen-Netherlands | 52.09; 4.25 | Anon, 1940 in Clapp and Kenk, 1963 |
|  | Plymouth-England | 50.86; -4.13 | Hall and Saunders, 1967 |
|  | Torbay-England | 50.45; -3.52 | Turner, 1966 |
|  | Angra do Heroísmo-Portugal | 38.65; -27-21 | Roch, 1940 |
|  | Funchal Madeira | 32.64;-16.91 | Roch, 1940 |
| *Nototeredo norvagica* (Spengler, 1792) | North of Porsangerfjorden-Norway | 70.60; 25.50 | Sneli, pers. com. (compiled by Brattegard, 2001) |
|  | Reykjavik harbour-Iceland | 64.15; -21.92 | Saemundson, 1903 in Clapp and Kenk, 1963 |
|  | Trondheim-Norway | 63.41; 10.41 | Dons, 1949; this study ( May 2002 to May 2003) |
|  | Bergen-Norway | 60.40; 5.31 | Nair, 1959 |

Table 2 (continued) Distribution of bivalve wood borers (Teredinidae) in European coastal waters.

| Species/ authority | Location | Coordinates | Source |
| --- | --- | --- | --- |
| *Nototeredo norvagica* (Spengler, 1792) | Hardanger Fjord-Norway | 60.16; 6.00 | Grieg, 1913 in Clapp and Kenk, 1963 |
|  | Odde-Norway | 60.06; 6.54 | Grieg, 1913 in Clapp and Kenk, 1963 |
|  | North Coast-Scotland | 58.22; -5.38 | Turner, 1966 |
|  | Carlisle-Southern Ireland | 54.90; -3.34 | Colgan, 1908 in Clapp and Kenk, 1963 |
|  | Borkum -Germany | 53.57; 6.66 | Grieg, 1913 in Clapp and Kenk, 1963 |
|  | Plymouth-England | 50.86; -4.13 | Lebour, 1946; Hall and Saunders, 1967 |
|  | Plymouth-Engand | 50.36;-4.15 | Harrington, 1922 |
|  | Toulindac-France | 47.60; -2.87 | This study (2009) |
|  | Berder-France | 47.55; -2.48 | This study (2009) |
|  | Penerf-France | 47.50; -2.61 | This study (2009) |
|  | Gulf of Trieste-Italy | 45.64; 13.75 | Graffe, 1902 in Clapp and Kenk, 1963 |
|  | Muggia-Italy | 45.60;13.75 | Roch, 1940 |
|  | Parec- Croatia | 45.22; 13.59 | Roch, 1940 |
|  | Omišalj-Croatia | 45.20; 14.55 | Roch, 1940 |
|  | Eupatoria-Ukraine | 45.18; 33.37 | Roch, 1940 |
|  | Funtana-Croatia | 45.17; 13.59 | Roch, 1940 |
|  | Canal di Leme-Italy | 45.13; 13.60 | Roch, 1940 |
|  | Rovinj-Croatia | 45.08; 13.63 | Cormio, 1947 in Clapp and Kenk, 1963; this study (May 2002 to May 2003) |
|  | Rovinj-Croatia | 45.08; 13.63 | Roch, 1940 |
|  | Fazana-Croatia | 44.92; 13.80 | Roch, 1940 |
|  | Brijuni, Pula-Croatia | 44.91; 13.76 | Roch, 1940 |
|  | Pula-Croatia | 44.86; 13.84 | Roch, 1940 |
|  | Alushta- Ukraine | 44.67; 34.41 | Roch, 1940 |
|  | Lussinpiccolo-Croatia | 44.53; 14.46 | Roch, 1940 |
|  | Jalta-Ukraine | 44.49; 34.16 | Roch, 1940 |
|  |  |  |  |

Table 2 (Continued). Distribution of *Teredo navalis* in European coastal waters.

| Species/ authority | Location | Coordinates | Source |
| --- | --- | --- | --- |
| *Nototeredo norvagica* (Spengler, 1792) | Zadar-Croatia | 44.11; 15.22 | Roch, 1940 |
|  | Viareggio-Italy | 43.88; 10.23 | Roch, 1940 |
|  | Monaco harbour-Monaco | 43.73; 7.42 | Roch, 1940 |
|  | Ancona-Italy | 43.62; 13.50 | Roch, 1940 |
|  | Livorno-Italy | 43.54: 10.30 | Roch, 1940 |
|  | Split-Croatia | 43.50; 16.44 | Roch, 1940 |
|  | Santander-Spain | 43.46; -3.79 | Roch, 1940 |
|  | Marseille-France | 43.29; 5.36 | Roch, 1940 |
|  | Komiža, Croatia | 43.04; 16.08 | Roch, 1940 |
|  | San Benedetto del Tronto-Italy | 42.95; 13.89 | Roch, 1940 |
|  | Lago di Varano-Italy | 42.80; 20.57 | Graffe, 1902 in Clapp and Kenk, 1963 |
|  | Lastovo-Croatia | 42.73; 16.88 | Roch, 1940 |
|  | Dubrovnik-Croatia | 42.65; 18.08 | Roch, 1940 |
|  | Banyuls-sur-Mer-France | 42.48; 3.12 | This study (2009) |
|  | Pescara-Italy | 42.46; 14.23 | Roch, 1940 |
|  | Vigo-Spain | 42.23; -8.73 | Roch, 1940 |
|  | San Domino-Italy | 42.11; 15.49 | Roch, 1940 |
|  | Termoli-Italy | 42.00; 14.99 | Roch, 1940 |
|  | Barcelona-Spain | 41.41; 2.23 | Giribet and Peñas, 1997 |
|  | Barcelona-Spain | 41.41; 2.23 | Roch, 1940 |
|  | Pasajes -Spain | 41.41; 2.22 | Benito Martinez, 1952 |
|  | Bari-Italy | 41.11; 16.87 | Roch, 1940 |
|  | Tarragona-Spain | 41.10; 1.25 | Roch, 1940 |
|  | Istanbul-Turkey | 41.01; 28.98 | Roch, 1940 |
|  | Naples-Italy | 40.84; 14.26 | Roch, 1940 |

Table 2 (Continued) Distribution of *Teredo navalis* in European coastal waters.

| *Species/ authority* | Location | Coordinates | Source |
| --- | --- | --- | --- |
| *Nototeredo norvagica* (Spengler, 1792) | Thessaloniki-Greece | 40.63; 22.93 | Roch, 1940 |
|  | Porto Conte-Italy | 40.59;8.21 | Jones et al., 1972 |
|  | Alghero, Sardinia-Italy | 40.56; 8.31 | Roch, 1940 |
|  | Taranto-Italy | 40.49; 17.31 | Roch, 1940 |
|  | Valona-Albania | 40.45; 19.48 | Graffe, 1902 in Clapp and Kenk, 1963 |
|  | Galliopoli-Italy | 40.05; 17.98 | Roch, 1940 |
|  | Volo-Greece | 39.36; 22.94 | Roch, 1940 |
|  | Lisbon-Portugal | 38.67; -9.20 | Borges et al., 2010 |
|  | Lipari, Messina-Italy | 38.47; 14.95 | Jones et al., 1972 |
|  | Mersin Bay-Turkey | 36.80; 34.64 | Pinar, 1979 in Bobat, 1995; this study (May 2002 to May 2003; 2007) |
|  | Kos-Greece | 36.75; 27.11 | Roch, 1940 |
|  | Iskenderun-Turkey | 36.59; 36.17 | Sen et al., 2010 |
|  | Rhodes-Greece | 36.44; 28.23 | Roch, 1940 |
|  | Chania, Kreeta-Greece | 35.51; 24.02 | Roch, 1940 |
|  | Larnaca-Cyprus | 34.79; 33.56 | Roch, 1940 |
|  | Funchal- Madeira | 32.64;-16.91 | Roch, 1940 |
| *Teredo bartschi* Clapp, 1923 | Olhão-Portugal | 37.00; -7.79 | this study ( May 2002 to May 2003) |
|  | Mersin Bay-Turkey | 36.80; 34.64 | this study ( May 2002 to May 2003) |
| *Bankia carinata* (Gray, 1827) | Trieste-Italy | 45.64; 13.75 | Graeffe, 1902 in Clapp and Kenk, 1963 |
|  | Rijeka-Croatia | 45.32; 14.44 | Roch, 1940 |
|  | Parec- Croatia | 45.22; 13.59 | Roch, 1940 |
|  | Funtana-Croatia | 45.17; 13.59 | Roch, 1940 |
|  | Canal di Leme-Italy | 45.13; 13.60 | Roch, 1940 |
|  | Rovinj-Croatia | 45.08; 13.63 | Cormio, 1947 in Clapp and Kenk, 1963; this study ( 2002, 2003) |
|  | Rovinj-Croatia | 45.08; 13.63 | Roch, 1940 |

Table 1 (continued) Distribution of bivalve wood borers (Teredinidae) in European coastal waters.

| *Species/ authority* | Location | Coordinates | Source |
| --- | --- | --- | --- |
| *Bankia carinata* (Gray, 1827) | Brijuni, Pula-Croatia | 44.91; 13.76 | Roch, 1940 |
|  | Dugi Otok-Croatia | 44.04; 15.02 | Roch, 1940 |
|  | Ancona-Italy | 43.62; 13.50 | Roch, 1940 |
|  | Komiža, Croatia | 43.04; 16.08 | Roch, 1940 |
|  | San Benedetto del Tronto-Italy | 42.95; 13.89 | Roch, 1940 |
|  | Guilianova-Italy | 42.75; 13.97 | Roch, 1940 |
|  | Lastovo-Croatia | 42.73; 16.88 | Roch, 1940 |
|  | Pescara-Italy | 42.46; 14.23 | Roch, 1940 |
|  | Termoli-Italy | 42.00;14.99 | Roch, 1940 |
|  | Vieste-Italy | 41.88; 16.17 | Roch, 1940 |
|  | Barcelona-Spain | 41.41; 2.23 | Giribet and Peñas, 1997 |
|  | Bari-Italy | 41.11; 16.87 | Roch, 1940 |
|  | Tarragona-Spain | 41.10; 1.25 | Roch, 1940 |
|  | Porto Conte-Italy | 40.59; 8,21 | Jones et al., 1972 |
|  | Messina-Italy | 38.19; 15.26 | Roch, 1940; Jones et al., 1972 |
|  | Mersin Bay-Turkey | 36.80; 34.64 | Bobat, 1995; this study (May 2002 to May 2003; 2007) |
|  | Iskenderun-Turkey | 36.59; 36.17 | Sen et al., 2010 |
| *Teredora malleolus* (Turton, 1822) | Vorran Island- UK | 57.15; -7.45 | EOL, 2007; accessed 14 May 2013 |
|  | Redcliff Point- UK | 50.61; -2.42 | EOL, 2007; accessed 14 May 2013 |
|  | Torbay- England | 50.45; -3.52 | Turner, 1966 |
|  | Angra do Heroísmo-Portugal | 38.65; -27-21 | Roch, 1940 |
|  | Messina-Italy | 38.19; 15.26 | Roch, 1940 |
| *Teredothyra dominicensis* (Bartsch, 1921) | Kas-Turkey | 37.03; 27.41 | Müller, 2011; Borges et al., 2012 |

**References**

Bavendammm W: *Die Holzbohrmuscheln oder Terediniden: ihre Lebensweise, Schädlicheit und Bekämpfung*: *Merkbl des Reichsinst fur Forst- und Holzwirtschaft, Reibe;* 1948.

Becker G: **Die bohrmuschel *Teredo* der gefährlichste holz-zestörer an den deutschen küsten**. *Holz als Roh-und Werkstoff* 1938, **1**(7): 251-254

Benito Martinez J: *Conseración de maderas en sus aspectos teórico, industrial y económico*: Instituto Forestal de investigatciones y experiencias, Madrid; 1952

Bobat A: **Marine wood-borer test with preservatives on the coast of Turkey**. In *Proceedings* *of the* *International Research Group on Wood Preservation IRG/WP/95-10091;* 1995:7pp.

Borges LMS, Valente AA, Palma P, Nunes L: **Changes in the wood boring community in the Tagus Estuary: a case study**. *Marine*

*Biodiversity Records*, 2010, *3*(e41): 1–7.

Borges LMS, Sivrikaya H, le Roux A, Shipway JR, Cragg SM, Costa FO: **Investigating the taxonomy and systematics of marine wood**

**borers (Bivalvia : Teredinidae) combining evidence from morphology, DNA barcodes and nuclear locus sequences**. *Invertebrate*

*Systematics* 2012, **26**(6): 572–582.

Brown CJ: ***The impact of copper-chrome-arsenic (CCA) wood preservatives on non-target marine organisms***. *PhD thesis*. Portsmouth

University, School of Biological Sciences; 1998.

Caspers H: **Der tierische Befall am Holz der Helgoländer** **Seebrücke**. *Zolol Anzeiger* 1941, **136**(1-2): 1-8

Clapp WF, Kenk R: *Marine borers an annotated bibliography*. Washington DC: Office of Naval Research Department of the Navy; 1963.

Coughlan J: *Wood-borer survey, Southamptom Water 1976, with proposals for future monitoring.* Laboratory note: Central Electricity Research Laboratories, (RD-L-N 145-77); 1977.

Dons C: **Marine boreorganismer XIII. *Teredo*- produksjonen og sjotemperaturen**. *Det Kongelige Norske Vindenskabers Selskab* 1949, **XXI** (42): 186–190.

Encyclopedia of Life [http://www.eol.org].

Giribet G, Peñas A: **Fauna malacológica del litoral del Garraf** **(NE de la Peninsula Ibérica)**. *Iberus* 1997, **15**(1): 41–93.

Hall GS, Saunders RG: *Incidence of marine borers round Britain’s coasts*. Timber Research and Development Association (TRADA); 1967.

Harrington CR: *Report of work done at the Marine Biological Station, Plymouth.* Rep Comm Inst Civil Engin, London;1922.

Hayes GP: *H. M. dockyard, Devonport: report onthe deterioration of structures exposed to sea action*. Rep Comm Inst Civil Engin, London;

1922.

Henningsson B, Norman E: *A marine borer test with water-borne preservatives.* In *Proceedings* *of the* *International Research Group on Wood Preservation IRG/WP/80-452;* 1980:5pp.

Henry E: **Invasions du taret dans le port de Marseille; moyens preservatifs.** *Bull de la Soc des Sci Nancy* 1909, **3**(10): 211–220.

Hoppe K: ***Teredo navalis*- the cryptogenic shipworm**. In i*nvasive aquatic species in Europe, distribution, impacts and management.* Edited by Leppäkoski E, Gollash S. Kluwer Academic Publishers; 2002: 116-119.

Jaeckel S: **Zur Kenntnis der marinen Molluskenfauna von Büsum (Holstein)**. *Zool Anzeiger* 1928, **79**(5-6): 134–142.

Jones EBG, Turner RD, Furtado SEJ, Kühne H: **Results of an international cooperative research programme on the biodeterioration of timber submerged in the sea**. *Mater Organismen* 1972, **7**: 93–118.

Kristensen E: **Attacks by *Teredo navalis* L. in the inner Danish waters in relation to environmental factors.** *Vidensk Meddr Dansk Naturh Foren* 1969, **132**: 199–210.

Lebour MV: **The species of *Teredo* from Plymouth waters**. *J Mar Biol Ass UK* 1946, **26**: 381–389.

Lebour MV: **Notes on the breeding of some lamellibranchs from Plymouth and their larvae**. *J Mar Biol Ass UK* 1938, **23**(1): 119–144.

Leege O: **Die Mollusken in der Umwelt des Ostfriesischen Inseln, ihre Erhaltung und ihr Lebenskamf.** *Aus der Heimat* 1939, **52**(7-8): 193–204.

Leloup E, Miller O: **La flore et la faune du bassin de chasse d´Ostend**. *Mém du Mus. royal d´His nat Belgique* 1940, **94**:1- 123.

Moll F: **Das Verhalten ungeschützter Hölzer gegen die Bohrmuschel**. *Kolonialforstliche Mitt der Zeitschr für Weltforstwirtschaft* 1940, **3**(4):

288–302.

Moll F, Roch F: **Die Holzbohrmuscheln und ihre Bekämpfung**. *Mitt der Ges für Vorratsschutz* 1929, **5** (3): 34–39.

Müller J: **Holzarten im historischen Schiffbau und ihre Gefährdung durch Terediniden**. *Skylliis* 2011, **10**(1): 90–94.

Nair NB: **Ecology of marine fouling and wood-boring organisms of western Norway**. *Sarsia* 1962, **8:** 1-88.

Nair NB: **The marine timber boring molluscs and crustaceans of W. Norway**. *Publs Biol Stn Espegrend Univ Bergen Arb Natun rekk.*

1959, **1**: 3–23.

Newell TM: *Liverpool and Birkenhead*. Rep Comm Inst Civil Engin, London; 1920: 109-115.

Norman E: **The geographical distribution and the growth of the wood-boring molluscs *Teredo navalis* L., *Psiloteredo megotara* (Hanley)**

**and *Xylophaga dorsalis* (Turton) on the Swedish west coast**. *Ophelia* 1977a, **16**: 233–250.

Norman E: T**he time of settlement on the Swedish West Coast of the woodboring Molluscs *Teredo navalis*, *Psiloteredo megotara* and**

***Xylophaga dorsalis***. *Material und Organismen* 1977b, **3**: 531–542.

Paalvast P, Velde G van der: **New threats of an old enemy: the distribution of the shipworm *Teredo navalis* L. (Bivalvia: Teredinidae)**

**related to climate change in the Port of Rotterdam area, the Netherlands**. *Mar Pollut Bull* 2011, **62**(8): 1822–1829.

Palma P: **Monitoring shipwreck sites**.  *Int J Naut Archaeol*, 2005, **342**: 323–331.

Praël A: *Evaluation of the efficacy and environmental impact of RH287 used as an anti-marine wood boring agent*. *PhD thesis*. Portsmouth

University, School of Biological Sciences; 2003.

Roch F: **Holzschädlinge des Meeres und deren Bekampfung**. *Zeitschr für hygien Zool* 1937, **29**(11): 305–316.

Roch F: **Die Terediniden des Mittelmeers**. *Thalassia* 1940, **4**: 1–147.

Santhakumaran LN, Sneli J-A: **Studies on the marine fouling and wood-boring organisms of the Trondheimsfjord (western Norway)**.

*Gunneria*,1984, **47**, 7–30.

Schwarz A: **Der Lichteinfluss auf die Fortbewegung, die Einregelung und das wachstum bei einingen niederen Tieren (*Littorina*,**

***Cardium*, *Mytilus*, *Balanus*, *Teredo*, *Sabellaria)***. *Senckenbergiana* 1932, **14**(6): 429–454.

Scott H: **Marine borers at Keyham**. *Trans royal Scottish arboricult Soc* 1910, **23**(2): 1- 203.

Sen S, Sivrikaya H, Yalcin M, Bakir AK, Ozturk B: **Fouling and boring organisms that deteriorate various European and tropical woods**

**at Turkish seas**. *African Journal of Biotechnology* 2010, **9**(17): 2566–2573

Sordyl H, Bönsch R, Gercken J, Gosselck F, Kreuzberg M, Schulze H: **Verbreitung und reproduktion des schiffsbohrwurms *Teredo navalis***

**L. and der küste Mercklenburg-Vorpommerns**. *Deutsche Gewässekundliche Mitteilungen*, 1998, **42**(4): 142–149.

Troschel E: **Ein neuer Feind unserer Wasserbauhölzer**. *Zentralbl der Bauverwaltung*, 1913, **33(**41): 273–274.

Tuente U, Piepenburg D, Spindler M: **Occurrence and settlement of the common shipworm *Teredo navalis* (Bivalvia: Teredinidae) in**

**Bremerhaven harbours, northern Germany**. *Helgol Mar Res* 2002, **56**(2): 87–94.

Turner RD: *A survey and illustrated catalogue of the Teredinidae:* The Museum of Comparative Zoology, Harvard University: 1966.

Windolf: **Der Bohrwurmbefall an Hafenbauten in den Cuxhavener Hafen**. *Wert-Reederei-Hafen* 1936, **17**(24): 404–408.
